# Inflammatory mediator polymorphisms associate with initial periodontitis in adolescents

**DOI:** 10.1002/cre2.40

**Published:** 2016-09-30

**Authors:** Anna Maria Heikkinen, Kaisa Kettunen, Leena Kovanen, Jari Haukka, Jessica Elg, Heidi Husu, Taina Tervahartiala, Pirkko Pussinen, Jukka Meurman, Timo Sorsa

**Affiliations:** ^1^ Department of Oral and Maxillofacial Diseases University of Helsinki and Helsinki University Hospital Helsinki Finland; ^2^ FIMM, Institute for Molecular Medicine Helsinki Finland; ^3^ Department of Health National Institute for Health and Welfare Helsinki Finland; ^4^ Department of Public Health, Clinicum University of Helsinki Helsinki Finland; ^5^ Division of Periodontology, Department of Dental Medicine Karolinska Institutet Huddinge Sweden

**Keywords:** adolescent, genetic, periodontitis, polymorphism

## Abstract

Several studies have addressed cytokine gene polymorphisms and their possible associations with periodontitis. We examined the association between salivary anti‐ and pro‐inflammatory mediator polymorphisms and initial periodontitis in Finnish adolescents, taking into account the effect of smoking. Salivary samples of 93 clinically examined adolescents from Eastern Finland were analyzed. Their oral health and smoking habits were recorded. Periodontal probing depth (PPD), and bleeding on probing (BOP) at four sites per tooth, root calculus (RC), and visible plaque index (VPI) were recorded from the index teeth. Salivary MMP‐8 median values were assessed. The sites with ≥4 mm PD were categorized as follows: PPD1 = one or more ≥4 mm pocket, PPD2 = two or more ≥4 mm pockets, and PPD3 = three or more ≥4 mm pockets. Genomic DNA was extracted from 300 μl of the saliva samples by genomic QIAamp® DNA Blood Mini Kit and genotyped for polymorphisms. Genetic variants for genotyping were selected from the following genes of interest: *S100A8, FCGR2A, FCGR2B, IL10, MMP8, MMP3, MMP13, VDR, TLR4, MMP2, MPO, ELANE, IL1A, IL1B, IL1RN, CD28, MMP9, DDX39B, NFKBIL1, LTA, TNF, SOD2, IL6, TLR4, TIMP1*, and *SYN1*. After false discovery rate control (FDR), polymorphisms in *MMP3* (rs679620, rs520540, rs639752), *CD28* (rs3116496), and *VDR* (rs2228570) associated (FDR q < 0.05) with deepened periodontal pockets. Smoking did not affect the results. Genetic polymorphisms of pro‐inflammatory mediators *MMP3*, *CD28*, and *VDR* seem to link to initial periodontitis.

Periodontitis is a complex chronic low‐grade infection‐induced tissue destruction inflammatory disease involving environmental factors such as smoking. The potential periodontal pathogens activate matrix metalloproteinases (MMPs), pro‐inflammatory cytokines, and their regulators together with enhanced inflammatory burden (Sorsa et al., 2016). The pathophysiology of periodontitis is associated with variations in multiple disease modifying genes. The environmental factors may thus interact with genetics (Laine, Crielaard, & Loos, 2012), and smoking as a confounding factor needs to be taken into account when assessing the effect of immune system and host response in periodontitis patients.

Several studies have been published regarding cytokine gene polymorphisms and their possible associations with periodontitis pathogenesis, when attempting to explain the complex nature of this disease. MMP polymorphisms have been of interest because of the essential role of MMPs in periodontal tissue remodeling and destruction. MMP‐8 (neutrophil collagenase) is the most abundant and important biomarker in periodontitis reflecting the initiation and progression of the disease. Increased MMP‐8 levels reflect the severity and course of periodontitis (Sorsa et al., 2016; Sorsa, Tjäderhane, & Salo, 2004; Sorsa et al., 2006). In smokers, salivary MMP‐8 concentrations are usually lower than in non‐smokers, even in adolescents (Liede et al., 1999; Heikkinen et al., 2010).

The roles of MMP‐2, MMP‐8, MMP‐9, and MMP‐12 have been analyzed with respect to the genetic polymorphisms; they may contribute to the susceptibility to periodontitis. MMP3 and tissue inhibitor of MMPs (TIMP1) SNPs have also been considered as possible candidates in the pathogenesis of periodontitis (Letra et al., 2012; Emingil et al., 2014). However, regarding matrix metallopeptidase 9 (*MMP9*) gene polymorphism, the results are ambivalent. Gürkan et al. (2008) reported that carrier status of *MMP9* ‐1562 T allele was lower in healthy Turkish subjects than in chronic periodontitis group (Gürkan et al., 2008). However, *MMP*9 ‐1562C/T was not related to the susceptibility to chronic periodontitis according to Holla, Fassmann, Muzík, Vanek, and Vasku (2006) and de Souza et al. (2005). Interleukins (IL) are pro‐inflammatory cytokines expressed mostly by leukocytes. Interleukin‐6 (IL‐6) is secreted by various cell types and has been shown to be important pro‐ and anti‐inflammatory mediator involved also in the pathogenesis of periodontitis by regulating the expression of IL‐1 and tumor necrosis factor alpha (TNF‐α) (Opal & DePalo, 2000). It also stimulates osteoclast differentiation as well as enhances B‐ and T‐cell growth, differentiation, and bone resorption (Hughes, Turner, Belibasakis, & Martuscelli, 2006). An association between the carriage rates of the *IL‐1* *, Alpha* *(IL1A*‐889, rs1800587), and periodontitis has been demonstrated (Laine, Loos, & Crielaard, 2010). Other salivary inflammatory biomarker candidate genes for periodontitis are considered, such as myeloperoxidase (*MPO*‐463G/A) (Erciyas, Pehlivan, Sever, & Orbak, 2010) and calcium‐binding protein A8 *(S100A8*) (Sun et al., 2011), Vitamin D (1,25‐Dihydroxyvitamin D3) receptor (*VDR*) *(*Chen, Li, Zhang, & Wang, 2012 *;* Laine et al., 2012 *),* toll‐like receptor 2, 4 (Folwaczny, Glas, Török, Limbersky, & Folwaczny, 2004; Schröder & Schumann, 2005), and lymphotoxin alpha (*LTA*) (Vasconcelos et al., 2012). However, studies on these cytokine and inflammatory mediator polymorphisms are rare, and the results are contradictory.

In Finland, 48% of the 30‐ to 34‐year‐olds have periodontitis (Suominen‐Taipale et al., 2008). According to the thesis by Heikkinen (2011) and Heikkinen et al. (2008), 56% of one birth cohort aged 15‐ to 16‐year‐old adolescents had more than one ≥4 mm periodontal pockets (Heikkinen, 2011; Heikkinen et al., 2008). Taking into account attachment loss and bleeding on probing, 10% of the subjects had initial periodontitis, and most of them were smokers (Heikkinen, 2011; Heikkinen et al., 2008). Heikkinen et al. (2011, 2010, 2012 also observed that smokers (25% of all participants) frequently harbored more periodontal bacteria than non‐smokers (66%), and smoking significantly decreased the values of the salivary biomarkers MMP‐8 and polymorphonuclear leukocytes (PMN) elastase in boys (Heikkinen et al., 2010; Heikkinen, 2011; Heikkinen et al., 2012).

The aim of the present study was to examine the association between the polymorphisms in salivary anti‐ and pro‐inflammatory mediators and initial periodontitis. We hypothesized that the salivary anti‐ and pro‐inflammatory mediator polymorphisms are associated with initial periodontitis.

## 1 MATERIAL AND METHODS

### 1.1 Study participants

**Figure 1:**
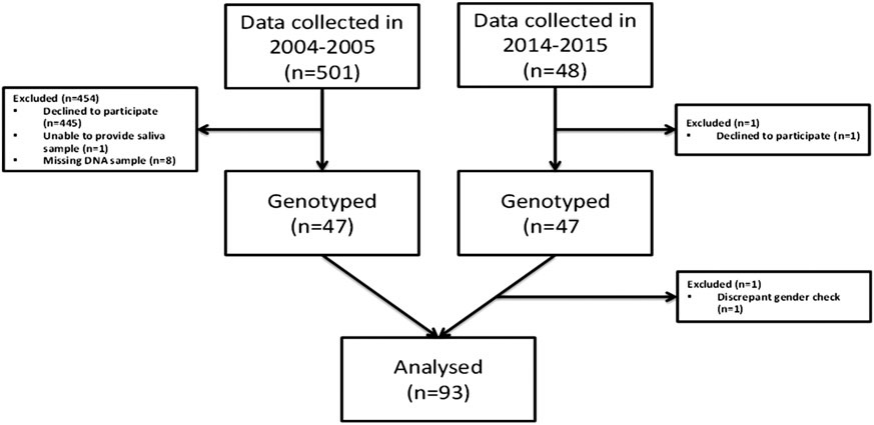
Study flow of the participants

Data were collected at the Kotka Health Center in Eastern Finland. The study was approved by the Ethical Committee of the Helsinki and Uusimaa Hospital District (Dnro 260/13/03/00/13). All the participants gave written informed consent. This study includes data from two Finnish adolescent birth cohorts collected at the Kotka Health Center in Eastern Finland in 2004–2005 and 2014–2015. Altogether, there were 94 participants for whom we had available saliva and DNA samples and who gave their approval for the saliva and DNA analyses (see Figure 1 for study flow of the participants).

Oral health of subjects was clinically examined in both cohorts. No periodontal therapy has been performed in the last recent year among these participants before this study, and after diagnostic procedures, they were treated by specialist in clinical periodontics. In short, periodontal parameters were recorded according to the WHO recommendations (World Health Organization, International Statistical Classification of Diseases and Related Health Problems, [Link]). Periodontal probing depth (PPD), bleeding on probing (BOP), and visible plaque index (VPI) were recorded at four sites per tooth, and all sites with ≥4 mm PD were recorded and categorized as follows: PPD1 = one or more ≥4 mm pocket, PPD2 = two or more ≥4 mm pockets, and PPD3 = three or more ≥4 mm pockets (Heikkinen et al., 2016). The cut‐point of the BOP value was 20 percentage meaning as gingivitis (Heikkinen, 2011; Heikkinen et al., 2008). Root calculus (RC) and VPI were recorded from index teeth.

Smoking habits (cigarettes per week indicated regular smoking) and pack‐years were recorded (Heikkinen et al., 2012). Stimulated salivary samples were collected and centrifuged, and the supernatants were used for the enzyme measurements, such as MMP‐8 analysis. MMP‐8 levels were analyzed by immunofluorometric assay (Medix Biochemica, Kauniainen, Finland). The interassay coefficient of variation (CV)% for this study was 6.3%, and detection limit for the assay was 0.08 μg/l (Heikkinen et al., 2010).

### 1.2 Candidate gene and SNP selection

Genetic variants for genotyping were selected from the following genes of interest: *S100A8, Fc fragment of IgG, low affinity IIa, receptor (FCGR2A), Fc fragment of IgG, low affinity IIb, receptor (FCGR2B), prostaglandin‐endoperoxide synthase 2 (PTGS2), IL10, MMP8, MMP3, MMP13, VDR, tumor necrosis factor (TNF; ligand) superfamily, Member 11 (TNFSF11), toll‐like receptor 4 (TLR4), MMP2, MPO, elastase, neutrophil expressed (ELANE), IL‐1, IL1A, Beta (IL1B), interleukin 1 receptor antagonist (IL1RN), CD28 molecule (CD28), MMP9, DEAD (Asp‐Glu‐Ala‐Asp) box polypeptide 39B (DDX39B), nuclear factor of Kappa light polypeptide gene enhancer in B‐cells inhibitor‐like 1 (NFKBIL1), LTA, TNF, Superoxide Dismutase 2, Mitochondrial (SOD2), IL6, TIMP metallopeptidase inhibitor 1 (TIMP1)*, and *Synapsin I (SYN1).* The majority of SNPs were selected based on previous publications. Some of the originally selected SNPs failed the Sequenom iPLEX design because of technical reasons. For these genes (*ELANE, IL1B, IL1RN, DDX39B*), replacing SNPs were selected from the HapMap database (http://www.hapmap.org) by use of the tag SNP selection algorithm Tagger included in the Haploview 4.2 software (Nature, 2003; Barrett, Fry, Maller, & Daly, 2005). Taq SNP selection was used in order to select optimal SNPs covering most of the genetic variation in the genomic area of interest. Flanking areas of the size of 10 kb were included both upstream and downstream of the actual gene encoding regions. Taq SNPs were selected based on the criteria of minor allele frequency (MAF) >0.05 in European population and pair‐wise r2 threshold of 0.9. Based on these selection criteria, 74 SNPs were successfully included in three genotyping multiplexes. Three SNPs (rs3795391, rs2125685, and rs2544480) were excluded during the optimization phase of genotyping. Table 1 gives all the SNPs successfully genotyped in this study.

**Table 1:** Successfully genotyped SNPs

Marker	Chr. location (GRCh37)	Gene	Alleles (A1/A2)	A1A1 (*n*, %)	A1A2 (*n*, %)	A2A2 (*n*, %)	MAF, %	HWE *p*‐value
rs3806232	1:153364130	*S100A8*	*T/C*	78 (85.7)	10 (11.0)	3 (3.3)	8.8	0.02
rs1801274	1:161479745	*FCGR2A*	*T/C*	23 (25.6)	50 (55.6)	17 (18.9)	46.7	0.40
rs5275	1:186643058	*PTGS2*	*T/C*	39 (45.4)	39 (45.4)	8 (9.3)	32.0	0.81
rs689466	1:186650751	*PTGS2*	*A/G*	59 (64.8)	30 (33.0)	2 (2.2)	18.7	0.73
rs1800872	1:206946407	*IL10*	*C/A*	52 (56.5)	36 (39.1)	4 (4.4)	23.9	0.58
rs1800871	1:206946634	*IL10*	*C/T*	52 (56.5)	36 (39.1)	4 (4.4)	23.9	0.58
rs1800896	1:206946897	*IL10*	*A/G*	29 (31.5)	46 (50.0)	17 (18.5)	43.5	1.00
rs1800587	2:113542960	*IL1A*	*C/T*	48 (52.2)	35 (38.0)	9 (9.8)	28.8	0.46
rs3917365	2:113586469	*IL1B*	*C/T*	79 (86.8)	11 (12.1)	1 (1.1)	7.1	0.37
rs1143633	2:113590467	*IL1B*	*G/A*	45 (49.5)	43 (47.3)	3 (3.3)	26.9	0.07
rs16062	2:113591081	*IL1B*	*C/T*	92 (100)			0.0	
rs1143627	2:113594387	*IL1B*	*T/C*	35 (38.5)	43 (47.3)	13 (14.3)	37.9	1.00
rs16944	2:113594867	*IL1B*	*G/A*	35 (39.3)	42 (47.2)	12 (13.5)	37.1	1.00
rs4251961	2:113874467	*IL1RN*	*T/C*	42 (46.2)	40 (44.0)	9 (9.9)	31.9	1.00
rs4251985	2:113877413	*IL1RN*	*G/T*	48 (52.8)	29 (31.9)	14 (15.4)	31.3	0.02
rs3213448	2:113879297	*IL1RN*	*G/A*	62 (67.4)	26 (28.3)	4 (4.4)	18.5	0.50
rs315935	2:113881365	*IL1RN*	*A/G*	85 (100)			0.0	
rs4251998	2:113881962	*IL1RN*	*G/A*	88 (100)			0.0	
rs3181051	2:113884673	*IL1RN*	*C/T*	87 (94.6)	5 (5.4)	0	2.7	1.00
rs3087263	2:113885768	*IL1RN*	*G/A*	85 (93.4)	5 (5.5)	1 (1.1)	3.9	0.11
rs3087266	2:113889100	*IL1RN*	*C/T*	55 (60.4)	28 (30.8)	8 (8.8)	24.2	0.15
rs4252022	2:113890161	*IL1RN*	*G/A*	89 (98.9)	1 (1.1)	0	0.6	1.00
rs315952	2:113890304	*IL1RN*	*T/C*	41 (44.6)	32 (34.8)	19 (20.7)	38.0	0.01
rs3116496	2:204594512	*CD28*	*T/C*	66 (72.5)	21 (23.1)	4 (4.4)	15.9	0.23
rs160690	6:2799060	*DDX39B*	*G/T*	56 (61.5)	30 (33)	5 (5.5)	22.0	0.76
rs7766569	6:2807359	*DDX39B*	*A/G*	52 (61.2)	32 (37.7)	1 (1.2)	20.0	0.17
rs5004021	6:2807721	*DDX39B*	*C/T*	90 (98.9)	1 (1.1)	0	0.6	1.00
rs12525298	6:2810889	*DDX39B*	*C/T*	53 (57.6)	35 (38.0)	4 (4.4)	23.4	0.77
rs186396	6:2811850	*DDX39B*	*A/G*	23 (25.3)	57 (62.6)	11 (12.1)	43.4	0.01
rs9405587	6:2812175	*DDX39B*	*T/G*	55 (59.8)	33 (35.9)	4 (4.4)	22.3	1.00
rs4084090	6:31218835	*Intergenic Chr. 6*	*T/C*	86 (93.5)	5 (5.4)	1 (1.1)	3.8	0.11
rs2071591	6:31515799	*NFKBIL1*	*C/T*	38 (42.2)	44 (48.9)	8 (8.9)	33.3	0.48
rs2857708	6:31533606	*LTA*	*G/A*	64 (69.6)	27 (29.4)	1 (1.1)	15.8	0.46
rs2009658	6:31538244	*LTA*	*C/G*	62 (67.4)	29 (31.5)	1 (1.1)	16.9	0.45
rs1800629	6:31543031	*TNF*	*G/A*	70 (76.1)	22 (23.9)	0	12.0	0.35
rs2736189	6:31564728	*NCR3*	*C/T*	62 (68.1)	28 (30.8)	1 (1.1)	16.5	0.45
rs4880	6:160113872	*SOD2*	*T/C*	30 (32.6)	47 (51.1)	15 (16.3)	41.9	0.83
rs5746092	6:160114311	*SOD2*	*C/G*	43 (46.7)	41 (44.6)	8 (8.7)	31.0	0.81
rs2758343	6:160114572	*SOD2*	*G/T*	30 (32.6)	48 (52.2)	14 (15.2)	41.3	0.53
rs1800796	7:22766246	*IL6*	*G/C*	90 (97.8)	2 (2.2)	0	1.1	1.00
rs1800795	7:22766645	*IL6*	*G/C*	34 (37.8)	41 (45.6)	15 (16.7)	39.4	0.66
rs11536889	9:120478131	*TLR4*	*G/C*	78 (84.8)	13 (14.1)	1 (1.1)	8.2	0.46
rs3765620	11:102595492	*MMP8*	*A/G*	36 (39.1)	46 (50.0)	10 (10.9)	35.9	0.50
rs1320632	11:102596063	*MMP8*	*T/C*	81 (88.0)	11 (12.0)	0	6.0	1.00
rs11225395	11:102596480	*MMP8*	*G/A*	36 (39.1)	46 (50.0)	10 (10.9)	35.9	0.50
rs639752	11:102707339	*MMP3*	*T/G*	34 (37.4)	46 (50.6)	11 (12.1)	37.4	0.51
rs650108	11:102708787	*MMP3*	*G/A*	44 (47.8)	40 (43.5)	8 (8.7)	30.4	1.00
rs520540	11:102709425	*MMP3*	*G/A*	35 (38.0)	46 (50.0)	11 (12.0)	37.0	0.65
rs679620	11:102713620	*MMP3*	*G/A*	34 (40.5)	41 (48.8)	9 (10.7)	35.1	0.63
rs522616	11:102715048	*MMP3*	*A/G*	51 (56.0)	36 (39.6)	4 (4.4)	24.2	0.57
rs2252070	11:102826539	*MMP13*	*A/G*	25 (27.2)	52 (56.5)	15 (16.3)	44.6	0.21
rs7975232	12:48238837	*VDR*	*A/C*	22 (23.9)	54 (58.7)	16 (17.4)	46.7	0.14
rs1544410	12:48239835	*VDR*	*G/A*	47 (52.2)	34 (37.8)	9 (10.0)	28.9	0.45
rs2228570	12:48272895	*VDR*	*C/T*	39 (42.4)	40 (43.5)	13 (14.1)	35.9	0.65
rs9533156	13:43147671	*TNFSF11*	*C/T*	23 (26.1)	44 (50.0)	21 (23.9)	48.9	1.00
rs2277438	13:43155168	*TNFSF11*	*A/G*	56 (61.5)	31 (34.1)	4 (4.4)	21.4	1.00
rs498670	13:91763380	*TLR4*	*G/A*	30 (33.0)	49 (53.9)	12 (13.2)	40.1	0.28
rs243865	16:55511806	*MMP2*	*C/T*	47 (51.1)	38 (41.3)	7 (7.6)	28.3	1.00
rs2759	17:56348106	*MPO*	*A/G*	85 (92.4)	6 (6.5)	1 (1.1)	4.4	0.15
rs11575868	17:56352883	*MPO*	*G/A*	79 (85.9)	13 (14.1)	0	7.1	1.00
rs2856857	17:56357833	*MPO*	*C/T*	69 (75.0)	21 (22.8)	2 (2.2)	13.6	0.67
rs2243828	17:56358884	*MPO*	*A/G*	63 (69.2)	24 (26.4)	4 (4.4)	17.6	0.46
rs2243827	17:56358941	*MPO*	*G/T*	69 (75.8)	20 (22.0)	2 (2.2)	13.2	0.65
rs740021	19:852104	*ELANE*	*G/T*	88 (96.7)	3 (3.3)	0	1.7	1.00
rs17223045	19:855587	*ELANE*	*C/T*	90 (98.9)	1 (1.1)	0	0.6	1.00
rs17216656	19:856015	*ELANE*	*G/A*	92 (100)			0.0	
rs17576	20:44640225	*MMP9*	*A/G*	27 (30.0)	48 (53.3)	15 (16.7)	43.3	0.52
rs3787268	20:44641731	*MMP9*	*G/A*	55 (60.4)	34 (37.4)	2 (2.2)	20.9	0.34
rs1062849	X:47445999	*TIMP1*	*C/T*	92 (100)			0.0	
rs6520279	X:47448096	*SYN1*	*T/C*	50 (54.4)	17 (18.5)	25 (27.2)	36.4	0.00
rs5906435	X:47448410	*SYN1*	*C/T*	54 (58.7)	16 (17.4)	22 (23.9)	32.6	0.00

Chromosomal location (assembly Feb. 2009 (GRCh37/hg19), associated gene, alleles, genotype counts and frequencies, minor allele frequencies (MAF) and Hardy–Weinberg equilibrium (HWE) *p*‐values are given for each SNP.

### 1.3 DNA extraction and genotyping

All saliva samples in both cohorts were immediately frozen at −20°C and kept at −70°C until DNA extraction in both cohorts. DNA was extracted from 300 μl of the saliva using a genomic QIAamp® DNA Blood Mini Kit. Genotyping was performed using the Agena MassARRAY SNP genotyping system and iPLEX Gold assays (Agena Bioscience, San Diego, CA, USA). Allele discrimination is based on primer extension with single mass‐modified nucleotides followed by Matrix‐assisted laser desorption/ionization Time‐of‐Flight (MALDI‐TOF) mass spectrometry. All reactions were designed in multiplexes of up to 35 SNPs, by use of Assay Design v2.0 software (Agena Bioscience). Genotyping reactions were performed on 20 ng of dried genomic DNA in 384‐well plate format according to the manufacturer's recommendations. Concentrations of the extension primers were adjusted according to their mass and varied between 7 and 24.6 μM. The data was collected using the MassARRAY Compact System (Agena Bioscience), and the genotypes were identified using Typer 4 software (Agena Bioscience). For quality control reasons, the genotype calls were also checked manually and corrected when necessary. Genotyping quality was examined by a detailed QC procedure consisting of success rate checks, duplicated samples, gender check for X‐chromosomal markers, water controls, and Hardy–Weinberg Equilibrium (HWE) testing.

Genotyping success rates ranged from 90.3% to 98.9%. All duplicate samples gave concordant results. One subject was excluded because of discrepant gender check results. Table 1 gives the genotype and allele frequencies and HWE *p*‐values. Five SNPs turned out to be non‐polymorphic (rs16062, rs315935, rs4251998, rs17216656, and rs1062849). Three SNPs (rs5004021, rs4252022, and rs17223045) with MAF < 0.01 were excluded. Rs5906435 and rs6520279 are X‐chromosomal explaining their deviation from the HWE. All other SNPs were in HWE (*p* > 0.01). Finally, 93 subjects and 63 SNPs were included in the statistical analysis.

### 1.4 Statistical analysis

The association between dichotomic outcome variables (salivary MMP‐8 > 163 μg/l, median value, BOP > 20%, RC > median, PPD1, PPD2, and PPD3) and SNPs were modeled using logistic regression model. For continuous outcome (PPD total), linear regression model was used. We assumed additive effect of SNPs. For all outcomes, two models were calculated: unadjusted with SNP as only explanatory variable, and VPI, regular smoking (i.e., those who reported weekly smoking), and two separate occasions for birth cohorts adjusted model. All data analyses were carried out using R language (http://www.R‐project.org) with package “SNPassoc”(R Development Core Team. R: A Language and Environment for Statistical Computing. Vienna, Austria, 2011). P values for each variable separately were corrected using false discovery rate (FDR) correction (Benjamini & Hochberg, 1995; Glickman, Rao, & Schultz, 2014). FDR q‐values less than 0.05 were considered significant. Population stratification or relatedness was not addressed.

### 1.5 Comparison of allele frequencies between populations

Allele frequencies of the SNPs with statistically significant findings (rs679620, rs520540, rs639752, rs3116496, and rs2228570) were compared with allele frequencies given in the ExAC database (http://exac.broadinstitute.org/). Frequencies for SNP rs639752 were not found in the ExAC database, thus they were searched for HapMap‐CEU from dbSNP database (http://www.ncbi.nlm.nih.gov/SNP/). In addition, the frequencies were compared with American population (1000 genomes AMR (1000 Genomes Ad Mixed American, including populations: MXL, PUR, CLM, PEL) and HAPMAP‐MEX (Mexican ancestry in Los Angeles, California), obtained from dbSNP database (Table 2).

**Table 2:** Comparison of allele frequencies between populations for SNPs with significant findings

SNP	Allels (Major/minor)	MAF	ExAC	HapMap‐CEU	AMR	HapMap‐MEX	Letra et?al. (Brazil) #	Letra et?al. (US) #
rs679620	G/A	0.37 (A)	0.40	0.59	0.32	—	0.48	0.41
rs520540	G/A	0.37 (A)	0.39	0.57	0.34	—	0.49	0.43
rs639752	T/G	0.38 (G)	—	0.57	0.34	0.35	0.48	0.51
rs3116496	T/C	0.16 (C)	0.14	0.17	0.11	0.13		
rs2228570	A/G	0.34 (A)	0,36	0.41	0.48	0.52		

MAF = minor allele frequency, minor allele given in parentheses; ExAC frequency given for European (Finnish) population; HapMap‐CEU = Utah residents with northern and western European ancestry from the CEPH collection; AMR = ad mixed American population; Mexican ancestry in Los Angeles, California.

# Letra et?al. (2012), ref.no7.

## 2 RESULTS

Saliva MMP‐8 median values were calculated and appeared to be 163.1 μg/l. However, no statistical significant findings were observed between salivary MMP‐8 median values and SPNs, which were analyzed in this study (Table 1).

Of the subjects (*n* = 94), 18 were healthy (BOP < 20% and no ≥ 4 mm pockets), 15 subjects had gingivitis (BOP ≥ 20% and no ≥ 4 mm pockets), and 61 had one or more ≥4 mm pocket (PPD1). Fifty‐one subjects had two or more ≥4 mm pockets (PPD2) and 37 had three or more ≥4 mm pockets (PPD3). Table 3 gives the basic characteristics of the study population.

**Table 3:** General characteristics of the participants with non‐missing DNA sample (*n* = 94)

	*n*	%	Missing (*n*)			
Male gender	47	50				
Female gender	47	50				
MMP‐8 > 163 μg/l	30	52.6	37			
BOP > 20%	54	57.4				
RC > median 5.85	47	50				
Smoking regularly #	16	17				
PPD1	61	64.9				
PPD2	51	54.3				
PPD3	37	39.4				

	Min.	Max.	Median	Mean	SD	Missing (*n*)
Age	15	17	15	15.51	0.76	
MMP‐8 μg/l	2.42	1147	163.1	176.4	165.9	37
BOP%	0.6	80.4	32.1	32.82	24.6	
VPI %	0	95.8	27.55	31.67	24.6	
RC %	0	79.2	5.85	9.52	12.6	
PPD total	0	32	2	4	5.8	

# Smoking weekly.

BOP = bleeding on probing; RC = root calculus; PPD1 = one or more ≥4mm pocket; PPD2 = two or more ≥4 mm pockets; PPD3 = three or more ≥4 mm pockets; VPI = visible plaque index.

The following results were obtained on the genetic polymorphism and periodontal disease clinical parameters and biomarkers. After false discovery rate control (FDR, q < 0.05) only for PPD3 (three or more ≥4 mm pockets) and *MMP‐3* (rs679620, rs520540, and rs639752), PPD3 and *CD28* (rs3116496) and PPD3 and VDR (rs2228570) were statistically significant (q = 0.04, 0.04, 0.04, and 0.04, respectively). Smoking or VPI did not affect these results. The significant results for the adjusted model are given in Table 4, and all association results (all phenotypes and both models) are given in the Supporting Information.

**Table 4:** All significant (FDR q < 0.05) SNP associations between the analyzed SNPs and phenotypes of the VPI and weekly smoking adjusted model. Major alleles were used as reference

Phenotype	Gene	SNP	Allele	OR	Lower	Upper	*p*‐value	FDR q‐value
PPD3	*MMP3*	rs679620	*A*	0.22	0.09	0.57	0.0006	0.04
PPD3	*MMP3*	rs520540	*A*	0.31	0.14	0.69	0.0020	0.04
PPD3	*MMP3*	rs639752	*G*	0.34	0.15	0.73	0.0036	0.04
PPD3	*CD28*	rs3116496	*C*	0.20	0.06	0.64	0.0015	0.04
PPD3	*Vitamin D receptor*	rs2228570	*T*	2.85	1.39	5.83	0.0024	0.04

*P*‐value obtained from logistic regression corrected for multiple testing by false discovery rate (FDR).

PPD3 = three or more ≥4 mm pockets.

Allele frequencies of the five SNPs with statistically significant findings (rs679620, rs520540, rs639752, rs3116496, and rs2228570) were compared with allele frequencies in Finnish, European, and American populations (ExAC, HAPMAP‐CEU, 1000 genomes AMR and HAPMAP‐MEX). No major population specific allele differences were detected.

## 3 DISCUSSION

The main finding of this study was that three *MMP3 SNPs* (rs67620, rs520540, and rs639752) were associated as exposure agents with three or more at least 4‐mm deep periodontal pockets by the strict statistical analysis (FDR). Interestingly, the same SNPs have previously been significantly associated to chronic adult periodontitis (rs679620 in US Caucasians), rs520540 on a trend level in US Caucasians, and rs639752 in US Caucasians and Brazilians (Letra et al., 2012). Our results also showed that those with clinical signs of initial periodontitis were separated from the healthy ones using the same definitions as in our previous publications of the whole cohort (Heikkinen et al., 2010; Heikkinen, 2011; Heikkinen et al., 2008; Heikkinen et al., 2016).

Stromelysin‐1 (MMP‐3) is involved in periodontal extracellular matrix processing of the basal membrane as well as the MMP activation cascades (Sorsa et al., 2004; Uitto, Overall, & McCulloch, 2003). Fibroblasts, chondrocytes, and endothelial cells can produce MMP‐3 as a part of inflammatory tissue destruction cascades (Haerian, Adonogianaki, Mooney, Docherty, & Kinane, 1995; Alpagot, Bell, Lundergan, Chambers, & Rudin, 2001; Reynolds, Hembry, & Meikle, 1994). Increased MMP‐3 levels in gingival crevicular fluid reflecting the course and severity of periodontitis have also been reported by Toyman et al. (2015) in adult periodontitis patients (Toyman et al., 2015). Previously, Ding et al. (2015) reported in their meta‐analysis that *MMP3* ‐1171 5A/6A polymorphism (rs35068180, not studied here) could be associated with decreased risk of periodontitis in an Asian population (Ding et al., 2015); however, Itagaki et al. (2004) pointed that MMP‐3 gene promoter polymorphisms did not influence the susceptibility to periodontitis in Japanese patients (Itagaki et al., 2004).

Another finding of this study was that *CD28* SNP rs3116496 [also known as +17(T/C)] major allele T associated with PPD3 in the adolescents. Previously, the polymorphism did not show any association with periodontitis in adults (e Silva et al., 2013). However, among non‐smokers, they reported a higher frequency of the T^−^ (CC) genotype in aggressive periodontitis compared with chronic periodontitis.

Furthermore, we observed that *Vitamin D receptor* SNP rs2228570 (also known as *FokI)* major allele C (also known as F allele) was protective to PPD3. In line, Naito et al. (2007) reported that heterozygous Ff individuals had a lower risk of severe chronic periodontitis than individuals without the F allele (Naito et al., 2007). On the contrary, Li et al. (2008) reported that F allele increased the susceptibility of aggressive periodontitis in Chinese (Li et al., 2008) and Park, Nam, and Choi (2006) reported that CC genotype associated with increased risk for generalized aggressive periodontitis in Koreans (Park et al., 2006). However, several studies have also reported no association between rs2228570 and periodontis (Wang, Zhang, & Chen, 2015; Wang et al., 2009; El Jilani et al., 2015; Tachi et al., 2003).

Laine et al. (2012) pointed in their review article that an association could be found between periodontitis and haplotypes in the*IL4* and *IL6* and *VDR* genes (Laine et al., 2012). Earlier, Kornman et al. (1997) observed that “severe periodontitis patients were accounted for by either smoking or the IL‐1 genotype” (Kornman et al., 1997). In our study, no such effects could be seen; in our study only 17% were regular smokers, and thus this potential confounding factor could not be detected. In this respect, cytokine gene polymorphisms are observed to be quite ambivalent.

However, as a limitation of this study of adolescents, the sample size was quite small. Majority of the participants were lost because in Finland it is difficult to obtain permission from adolescents and their parents for a genetic study. This study has several strengths such as comprehensive oral health examination, unique age group, and adolescents. Despite the strengths, the limited sample size needs to be acknowledged.

To conclude, our study was the first to investigate in an adolescent population the genetic background of pathogenesis of initial or early periodontitis. We found that the genetic polymorphisms in *MMP3, CD28*, or *VDR* gene seem to be important in this respect, otherwise than *MMP8 gene*, as might be assumed (Holla, Hrdlickova, Vokurka, & Fassmann, 2012). However, more investigations are needed in larger materials for final conclusion. Further, new point‐of‐care chair‐side diagnostic tools have been developed to conveniently and consistently identify those adolescents with elevated risk for ongoing active gingivitis and periodontitis to be guided to regular examination and treatments (Heikkinen et al., 2016). Nevertheless, our study hypothesis was confirmed by showing an association between genetic polymorphism and periodontal disease parameters and markers.

## CONFLICT OF INTEREST

All authors certify that they have no affiliations with or involvement in any organization or entity with any financial interest or non‐financial interest in the subject matter or materials discussed in this manuscript.

## Supporting information

Table S1 Supporting info item

## References

[cre240-bib-0001] Alpagot, T. , Bell, C. , Lundergan, W. , Chambers, D. W. , & Rudin, R. (2001). Longitudinal evaluation of GCF MMP‐3 and TIMP‐1 levels as prognostic factors for progression of periodontitis. Journal of Clinical Periodontology, 28, 353–359. 11314892 10.1034/j.1600-051x.2001.028004353.x

[cre240-bib-0002] Barrett, J. C. , Fry, B. , Maller, J. , & Daly, M. J. (2005). Haploview: Analysis and visualization of LD and haplotype maps. Bioinformatics, 15(21), 263–265. 10.1093/bioinformatics/bth457 15297300

[cre240-bib-0003] Benjamini, Y. , & Hochberg, Y. (1995). Controlling the false discovery rate: A practical and powerful approach to multiple testing. J Royal Statistics Soc. Ser B (Methodological), 57, 289–300.

[cre240-bib-0004] Chen, L. L. , Li, H. , Zhang, P. P. , & Wang, S. M. (2012). Association between vitamin D receptor polymorphisms and periodontitis: A meta‐analysis. Periodontology 2000, 83, 1095–1103. 10.1902/jop.2011.110518 22181683

[cre240-bib-0005] de Souza, A. P. , Trevilatto, P. C. , Scarel‐Caminaga, R. M. , de Brito, R. B Jr. , Barros, S. P. , & Line, S. R. (2005). Analysis of the MMP‐9 (C‐1562 T) and TIMP‐2 (G‐418C) gene promoter polymorphisms in patients with chronic periodontitis. Journal of Clinical Periodontology, 32, 207–211. 15691353 10.1111/j.1600-051X.2005.00665.x

[cre240-bib-0006] Ding, C. , Chen, X. , Zhang, P. T. , Huang, J. P. , Xu, Y. , Chen, N. , & Zhong, L. J. (2015). Matrix metalloproteinase‐3 ‐1171 5A/6A polymorphism (rs35068180) is associated with risk of periodontitis. Scientific Reports, 5, 11667. 26123623 10.1038/srep11667 PMC4485030

[cre240-bib-0007] e Silva, M. R. , Moreira, P. R. , da Costa, G. C. , Saraiva, A. M. , de Souza, P. E. , Amormino, S. A. , … Dutra, W. O. (2013). Association of CD28 and CTLA‐4 gene polymorphisms with aggressive periodontitis in Brazilians. Oral Diseases, 19, 568–576. 23163888 10.1111/odi.12036

[cre240-bib-0008] El Jilani, M. M. , Mohamed, A. A. , Zeglam, H. B. , Alhudiri, I. M. , Ramadan, A. M. , Saleh, S. S. , … Enattah, N. S. (2015). Association between vitamin D receptor gene polymorphisms and chronic periodontitis among Libyans. Libyan J Med, 19(10), 216–222. 10.3402/ljm.v10.26771 PMC4368710 25795245

[cre240-bib-0009] Emingil, G. , Han, B. , Gürkan, A. , Berdeli, A. , Tervahartiala, T. , Salo, T. , … Sorsa, T. (2014). Matrix metalloproteinase (MMP)‐8 and tissue inhibitor of MMP‐1 (TIMP‐1) gene polymorphisms in generalized aggressive periodontitis: Gingival crevicular fluid MMP8 and TIMP‐1 levels and outcome of periodontal therapy. Journal of Periodontology, 85, 1070–1080. 24283658 10.1902/jop.2013.130365

[cre240-bib-0010] Erciyas, K. , Pehlivan, S. , Sever, T. , & Orbak, R. (2010). Genetic variation of myeloperoxidase gene contributes to aggressive periodontitis: a preliminary association study in Turkish population. Disease Markers, 28, 95–99. 20364045 10.3233/DMA-2010-0689 PMC3833700

[cre240-bib-0011] Folwaczny, M. , Glas, J. , Török, H. P. , Limbersky, O. , & Folwaczny, C. (2004). Toll‐like receptor (TLR) 2 and 4 mutations in periodontal disease. Clinical and Experimental Immunology, 135, 330–335. 14738464 10.1111/j.1365-2249.2004.02383.x PMC1808953

[cre240-bib-0012] Glickman, M. E. , Rao, S. R. , & Schultz, M. R. (2014). False discovery rate control is a recommended alternative to Bonferroni‐type adjustments in health studies. Journal of Clinical Epidemiology, 67, 850–857. 24831050 10.1016/j.jclinepi.2014.03.012

[cre240-bib-0013] Gürkan, A. , Emingil, G. , Saygan, B. H. (2008). Gene polymorphisms of matrix metalloproteinase‐2, ‐9 and ‐12 in periodontal health and severe chronic periodontitis. Archives of Oral Biology, 53, 337–345. 18155181 10.1016/j.archoralbio.2007.11.002

[cre240-bib-0014] Haerian, A. , Adonogianaki, E. , Mooney, J. , Docherty, J. P. , & Kinane, D. F. (1995). Gingival crevicular stromelysin, collagenase and tissue inhibitor of metalloproteinases levels in healthy and diseased sites. Journal of Clinical Periodontology, 22, 505–509. 7560232 10.1111/j.1600-051x.1995.tb00797.x

[cre240-bib-0015] Heikkinen, A. M. (2011). Oral health, smoking and adolescence. University of Helsinki, Faculty of Medicine, Institute of Dentistry. http://urn.fi/URN:ISBN:978‐952‐10‐7250‐5.

[cre240-bib-0016] Heikkinen, A. M. , Pajukanta, R. , Pitkäniemi, J. , Broms, U. , Sorsa, T. , Koskenvuo, M. , & Meurman, J. H. (2008). The effect of smoking on periodontal health of 15‐ to 16‐year‐old adolescents. Journal of Periodontology, 79, 2042–2047. 18980511 10.1902/jop.2008.080205

[cre240-bib-0017] Heikkinen, A. M. , Sorsa, T. , Pitkäniemi, J. , Tervahartiala, T. , Kari, K. , Broms, U. , … Meurman, J. H. (2010). Smoking affects diagnostic salivary periodontal disease biomarker levels in adolescents. Journal of Periodontology, 81, 1299–1307. 20450405 10.1902/jop.2010.090608

[cre240-bib-0018] Heikkinen, A. M. , Pitkäniemi, J. , Kari, K. , Pajukanta, R. , Elonheimo, O. , Koskenvuo, M. , & Meurman, J. H. (2012). Effect of teenage smoking on the prevalence of periodontal bacteria. Clinical Oral Investigations, 16, 571–580. 21340603 10.1007/s00784-011-0521-3

[cre240-bib-0019] Heikkinen, A. M. , Nwhator, S. O. , Rathnayake, N. , Mäntylä, P. , Vatanen, P. , & Sorsa, T. (2016). Pilot study on oral health status as assessed by an active matrix metalloproteinase‐8 chairside mouthrinse test in adolescents. Journal of Periodontology, 87, 36–40. 26430926 10.1902/jop.2015.150377

[cre240-bib-0020] Holla, L. I. , Fassmann, A. , Muzík, J. , Vanek, J. , & Vasku, A. (2006). Functional polymorphisms in the matrix metalloproteinase‐9 gene in relation to severity of chronic periodontitis. Journal of Periodontology, 77, 1850–1855. 17076610 10.1902/jop.2006.050347

[cre240-bib-0021] Holla, L. I. , Hrdlickova, B. , Vokurka, J. , & Fassmann, A. (2012). Matrix metalloproteinase 8 (MMP8) gene polymorphisms in chronic periodontitis. Archives of Oral Biology, 57, 188–196. 21920499 10.1016/j.archoralbio.2011.08.018

[cre240-bib-0022] Hughes, F. J. , Turner, W. , Belibasakis, G. , & Martuscelli, G. (2006). Effects of growth factors and cytokines on osteoblast differentiation. Periodontology 2000, 41, 48–72. 16686926 10.1111/j.1600-0757.2006.00161.x

[cre240-bib-0023] Itagaki, M. , Kubota, T. , Tai, H. , Shimada, Y. , Morozumi, T. , & Yamazaki, K. (2004). Matrix metalloproteinase‐1 and ‐3 gene promoter polymorphisms in Japanese patients with periodontitis. Journal of Clinical Periodontology, 31, 764–769. 15312099 10.1111/j.1600-051X.2004.00553.x

[cre240-bib-0024] Kornman, K. S. , Crane, A. , Wang, H. Y. , di Giovine, F. S. , Newman, M. G. , Pirk, F. W. , … Duff, G. W. (1997). The interleukin‐1 genotype as a severity factor in adult periodontal disease. Journal of Clinical Periodontology, 24, 72–77. 9049801 10.1111/j.1600-051x.1997.tb01187.x

[cre240-bib-0025] Laine, M. L. , Loos, B. G. , & Crielaard, W. (2010). Gene polymorphisms in chronic periodontitis. Int J Dent, 2010, 324719. 20339487 10.1155/2010/324719 PMC2844543

[cre240-bib-0026] Laine, M. L. , Crielaard, W. , & Loos, B. G. (2012). Genetic susceptibility to periodontitis. Periodontology 2000, 58, 37–68. 22133366 10.1111/j.1600-0757.2011.00415.x

[cre240-bib-0027] Letra, A. , Silva, R. M. , Rylands, R. J. , Silveira, E. M. , de Souza, A. P. , Wendell, S. K. , … Vieira, A. R. (2012). MMP3 and TIMP1 variants contribute to chronic periodontitis and may be implicated in disease progression. Journal of Clinical Periodontology, 39, 707–716. 22671570 10.1111/j.1600-051X.2012.01902.x PMC3393793

[cre240-bib-0028] Li, S. , Yang, M. H. , Zeng, C. A. , Wu, W. L. , Huang, X. F. , Ji, Y. , & Zeng, J. Q. (2008). Association of vitamin D receptor gene polymorphisms in Chinese patients with generalized aggressive periodontitis. Journal of Periodontal Research, 43, 360–363. 18205735 10.1111/j.1600-0765.2007.01044.x

[cre240-bib-0029] Liede, K. E. , Haukka, J. K. , Hietanen, J. H. , Mattila, M. H. , Rönkä, H. , & Sorsa, T. (1999). The association between smoking cessation and periodontal status and salivary proteinase levels. Journal of Periodontology, 70, 1361–1368. 10588500 10.1902/jop.1999.70.11.1361

[cre240-bib-0030] Naito, M. , Miyaki, K. , Naito, T. , Zhang, L. , Hoshi, K. , Hara, A. , … Nakayama, T. (2007). Association between vitamin D receptor gene haplotypes and chronic periodontitis among Japanese men. International Journal of Medical Sciences, 22(4), 216–222. 10.7150/ijms.4.216 PMC1975778 17848979

[cre240-bib-0031] International HapMap consortium (2003). The international HapMap project. Nature, 18(426), 789–796. HapMap database *(* *http://www.hapmap.org* *)* 10.1038/nature02168 14685227

[cre240-bib-0032] Opal, S. M. , & DePalo, V. A. (2000). Anti‐inflammatory cytokines. Chest, 117, 1162–1172. 10767254 10.1378/chest.117.4.1162

[cre240-bib-0033] Park, K. S. , Nam, J. H. , & Choi, J. (2006). The short vitamin D receptor is associated with increased risk for generalized aggressive periodontitis. Journal of Clinical Periodontology, 33, 524–528. 16899094 10.1111/j.1600-051X.2006.00944.x

[cre240-bib-0034] R Development Core Team . R: A Language and Environment for Statistical Computing. Vienna, Austria: 2011 http://www.R‐project.org/.

[cre240-bib-0035] Reynolds, J. J. , Hembry, R. M. , & Meikle, M. C. (1994). Connective tissue degradation in health and periodontal disease and the roles of matrix metalloproteinases and their natural inhibitors. Advances in Dental Research, 8, 312–319. 7865092 10.1177/08959374940080022701

[cre240-bib-0036] Schröder, N. W. , & Schumann, R. R. (2005). Single nucleotide polymorphisms of toll‐like receptors and susceptibility to infectious disease. The Lancet Infectious Diseases, 5, 156–164. 15766650 10.1016/S1473-3099(05)01308-3

[cre240-bib-0037] Sorsa, T. , Tjäderhane, L. , & Salo, T. (2004). Matrix metalloproteinases (MMPs) in oral diseases. Oral Diseases, 10, 311–318. 15533204 10.1111/j.1601-0825.2004.01038.x

[cre240-bib-0038] Sorsa, T. , Tjäderhane, L. , Konttinen, Y. T. , Lauhio, A. , Salo, T. , Lee, H. M. , … Mäntylä, J. (2006). Matrix metalloproteinases: Contribution to pathogenesis, diagnosis and treatment of periodontal inflammation. Annals of Medicine, 38, 306–321. 16938801 10.1080/07853890600800103

[cre240-bib-0039] Sorsa, T. , Gursoy, U. K. , Nwhator, S. , Hernandez, M. , Tervahartiala, T. , Leppilahti, J. , … Mäntylä, P. (2016). Analysis of matrix metalloproteinases, especially MMP‐8, in gingival creviclular fluid, mouthrinse and saliva for monitoring periodontal diseases. Periodontology 2000, 70, 142–163. 26662488 10.1111/prd.12101

[cre240-bib-0040] Sun, X. , Meng, H. , Shi, D. , Xu, L. , Zhang, L. , Chen, Z. , … Lu, R. (2011). Analysis of plasma calprotectin and polymorphisms of S100A8 in patients with aggressive periodontitis. Performance Research, 46, 354–360. 10.1111/j.1600-0765.2011.01350.x 21463326

[cre240-bib-0041] Suominen‐Taipale, L. , Nordblad, A. , Vehkalahti, M. , & Aromaa, A. (2008). Oral health in the finnish adult population. Health 2000 Survey. Publications of the National Public Health Institute B 25 / 2008.

[cre240-bib-0042] Tachi, Y. , Shimpuku, H. , Nosaka, Y. , Kawamura, T. , Shinohara, M. , Ueda, M. , … Ohura, K. (2003). Vitamin D receptor gene polymorphism is associated with chronic periodontitis. Life Sciences, 73, 3313–3321. 14572874 10.1016/j.lfs.2003.03.001

[cre240-bib-0043] Toyman, U. , Tüter, G. , Kurtiş, B. , Kıvrak, E. , Bozkurt, Ş. , Yücel, A. A. , & Serdar, M. (2015). Evaluation of gingival crevicular fluid levels of tissue plasminogen activator,plasminogen activator inhibitor 2, matrix metalloproteinase‐3 and interleukin 1‐β in patients with different periodontal diseases. Journal of Periodontal Research, 50, 44–51. 24690077 10.1111/jre.12179

[cre240-bib-0044] Uitto, V. J. , Overall, C. M. , & McCulloch, C. (2003). Proteolytic host cell enzymes in gingival crevice fluid. Periodontology 2000, 31, 77–104. 12656997 10.1034/j.1600-0757.2003.03106.x

[cre240-bib-0045] Vasconcelos, D. F. , da Silva, M. A. , Marques, M. R. , de Brito Júnior, R. B. , Vasconcelos, A. C. , & Barros, S. P. (2012). Lymphotoxin‐alpha gene polymorphism +252A/G (rs909253, A/G) is associated with susceptibility to chronic periodontitis: A pilot study. ISRN Dent, 2012, 617245. 23050158 10.5402/2012/617245 PMC3463161

[cre240-bib-0046] Wang, C. , Zhao, H. , Xiao, L. , Xie, C. , Fan, W. , Sun, S. , … Zhang, J. (2009). Association between vitamin D receptor gene polymorphisms and severe chronic periodontitis in a Chinese population. Journal of Periodontology, 80, 603–608. 19335080 10.1902/jop.2009.080465

[cre240-bib-0047] Wang, X. , Zhang, T. L. , & Chen, D. (2015). Lack of association between the vitamin D receptor polymorphism rs2228570 and chronic periodontitis in a Han Chinese population. Genetics and Molecular Research, 14, 12299–122305. 26505378 10.4238/2015.October.9.18

[cre240-bib-0048] World Health Organization, International Statistical Classification of Diseases and Related Health Problems , 10th Revision (2007). Available: http://www.who.int/classifications/apps/icd/icd10online/[2007, 2007, 09/14].

